# Ethyl Acetate Fractions of *Papaver rhoeas* L. and *Papaver nudicaule* L. Exert Antioxidant and Anti-Inflammatory Activities

**DOI:** 10.3390/antiox10121895

**Published:** 2021-11-26

**Authors:** Hail Kim, Sanghee Han, Kwangho Song, Min Young Lee, BeumJin Park, In Jin Ha, Seok-Geun Lee

**Affiliations:** 1 Graduate School, Kyung Hee University, Seoul 02447, Korea; khi3598@khu.ac.kr (H.K.); sanghee8757@khu.ac.kr (S.H.); 2 Korean Medicine Clinical Trial Center, Kyung Hee University Korean Medicine Hospital, Seoul 02454, Korea; siwcazb0@gmail.com (K.S.); papermint221@gmail.com (M.Y.L.); presto3477@gmail.com (B.P.)

**Keywords:** *Papaver nudicaule*, *Papaver rhoeas*, anti-inflammatory, antioxidant, STAT3, NF-κB, Nrf2

## Abstract

Abnormal inflammation and oxidative stress are involved in various diseases. *Papaver rhoeas* L. possesses various pharmacological activities, and a previously reported analysis of the anti-inflammatory effect of *P. nudicaule* ethanol extracts and alkaloid profiles of the plants suggest isoquinoline alkaloids as potential pharmacologically active compounds. Here, we investigated anti-inflammatory and antioxidant activities of ethyl acetate (EtOAc) fractions of *P. nudicaule* and *P. rhoeas* extracts in lipopolysaccharide (LPS)-stimulated RAW264.7 cells. EtOAc fractions of *P. nudicaule* and *P. rhoeas* compared to their ethanol extracts showed less toxicity but more inhibitory activity against LPS-induced nitric oxide production. Moreover, EtOAc fractions lowered the LPS-induced production of proinflammatory molecules and cytokines and inhibited LPS-activated STAT3 and NF-κB, and additionally showed significant free radical scavenging activity and decreased LPS-induced reactive oxygen species and oxidized glutathione. EtOAc fractions of *P. nudicaule* increased the expression of HO-1, GCLC, NQO-1, and Nrf2 in LPS-stimulated cells and that of *P. rhoeas* enhanced NQO-1. Furthermore, metabolomic and biochemometric analyses of ethanol extracts and EtOAc fractions indicated that EtOAc fractions of *P. nudicaule* and *P. rhoeas* have potent anti-inflammatory and antioxidant activities, further suggesting that alkaloids in EtOAc fractions are potent active molecules of tested plants.

## 1. Introduction

The inflammatory response protects the body against harmful external substances by secreting various inflammatory mediators by immune cells; however, an uncontrolled response can result in excessive cellular/tissue damage that leads to chronic inflammation and destruction of normal tissue [1]. The overproduction of pro-inflammatory mediators in chronic inflammation has been associated with the onset of chronic degenerative diseases, such as arthritis, atherosclerosis, asthma, Alzheimer’s disease, and others [1,2,3,4,5]. During inflammation, the macrophages mainly promote the production of pro-inflammatory mediators, including interleukin (IL)-1β, IL-6, tumor necrosis factor (TNF)-α, reactive oxygen species (ROS), nitric oxide (NO), and prostaglandin E2 (PGE2) [6].

Another key component of inflammation is oxidative stress, which reflects an imbalance in ROS, which reduces the production of antioxidant enzymes that protect tissues from oxidative damage [7,8]. In particular, the induction of phase 2 detoxifying enzymes and cytoprotective genes, such as heme oxygenase-1 (HO-1), glutamate-cysteine ligase catalytic (GCLC), and NAD(P)H quinone oxidoreductase 1 (NQO1), is mediated by nuclear factor erythroid-2-related factor 2 (Nrf2), which plays a central role in protecting cells from inflammatory and oxidative damage [9,10,11]. Therefore, developing anti-inflammatory agents and antioxidants that can control the activation of macrophages for preventing and treating various diseases is crucial.

*Papaver rhoeas* L. (*P. rhoeas*) and *Papaver nudicaule* L. (*P. nudicaule*) in the *Papaver* genus of *the Papaveraceae* family have been widely used for ornamentation owing to their multicolored flowers [12]. These plants have been treated as folk medicines for a long time. *P. rhoeas* has been used to treat inflammation, cough, diarrhea, respiratory problems, asthma, insomnia, and pain, and also consumed as food [13,14,15,16]. *P. nudicaule* has been used as a mild diaphoretic and a source of vitamin C [17,18]. *Papaver* species produce various alkaloids, particularly isoquinoline alkaloids, which have potent pharmacological activities such as narcotic analgesic, antimicrobial, cough suppressant, and anticancer effects [15,19,20,21,22]. Recent studies have also confirmed various pharmacological effects of *P. rhoeas* extracts, including antioxidant, cytoprotective, antimicrobial, antimutagenic, anti-analgesic, and antiulcerogenic activities [15,19,23,24,25,26,27]. However, few biological activities of *P. nudicaule* have been reported. We recently reported decreased response of lipopolysaccharide (LPS)-induced inflammation by ethanol (EtOH) extracts of *P. nudicaule* by inhibiting the signal transducer and activator of transcription 3 (STAT3) and nuclear factor-kappa B (NF-κB) pathways [28]. However, EtOH extracts of *P. rhoeas* did not show any effect on LPS-induced inflammation in RAW264.7 macrophage cells (data not shown). In addition, EtOH extracts of both Papaver species did not exhibit antioxidant activity in our system (data not shown). Although both *P. rhoeas* and *P. nudicaule* are known to produce pharmacologically active alkaloids, EtOH extraction might not be sufficient for biological activities. Therefore, in the current study, liquid-liquid extraction (LLE)-based ethyl acetate (EtOAc) fractions from EtOH extracts of *P. rhoeas* and *P. nudicaule* were prepared to enrich alkaloids, and their potential anti-inflammatory and antioxidant activities were examined.

## 2. Materials and Methods

### 2.1. Plant Materials and Sample Preparation

*P. nudicaule* and *P. rhoeas* were collected by the Korea National Academy of Agricultural Science, Rural Development Administration. The five cultivars of *P. nudicaule* and *P. rhoeas* samples were immediately freeze-dried after washing with clean water. The EtOH extractions of each ten-gram finely ground sample of aerial parts of lyophilized Papaver species were performed as previously described [28]. Each EtOH extract was thereafter suspended in distilled H_2_O and polar phase sequentially partitioned with n-hexane and EtOAc (Table 1).

### 2.2. Cell Culture and Reagents

RAW264.7 murine macrophage cells (Korean Cell Line Bank, Seoul, Republic of Korea) were cultured as previously described [28]. 3-(4,5-dimethylthiazol-2-yl)- and 5-diphenyl-tetrazolium bromide (MTT) and LPS (*Escherichia coli* serotype 0111: B4) were purchased from Sigma-Aldrich (St. Louis, MO, USA). 

### 2.3. Cell Viability Assays

MTT assays were performed to measure cell viability in RAW264.7 cells (1 × 10^4^ cells/well in 96-well plates) after treatment with EtOH extracts or EtOAc fractions of *P. nudicaule* and *P. rhoeas* for 24 h as previously described [28]. Data are presented as mean ± the standard error of the mean (SEM) from at least three independent experiments in triplicates.

### 2.4. Nitric Oxide Assays

A NO detection kit (iNtRON Biotechnology, Sungnam, Republic of Korea) was used for measuring the nitrite concentration in the culture supernatant as an indicator of NO production. NO assays in RAW264.7 cells (5 × 10^5^ cells/well in 6-well plates) were performed at least three independent experiments in triplicate according to the instructions of the manufacturer and also as previously described [28]. Data are presented as mean  ±  SEM.

### 2.5. 2,2-Diphenyl-1-picrylhydrazyl (DPPH) Free Radical Scavenging Activity

The free radical scavenging activity of the EtOAc fraction of *P. nudicaule* and *P. rhoeas* was evaluated using 2,2-diphenyl-1-picrylhydrazyl (DPPH) analysis. Briefly, 150 μL of 10 μM DPPH (Sigma Aldrich, St. Louis, MO, USA) was added to 150 μL of serial dilution samples, and ascorbic acid was used as a positive control because it is a well-known antioxidant. The mixture was then incubated at room temperature for 15 min in the dark. The absorbance of each sample solution was measured at 515 nm using a microplate reader. The free radical scavenging activity was calculated as follows: DPPH radical scavenging activity (%) = (A_blank_ − A_sample_)/A_blank_ × 100 (A_blank_: the absorbance of the DPPH solution and A_sample_: the absorbance of the test sample). Data are presented as the mean ± SEM from at least three independent experiments in triplicate.

### 2.6. GSH/GSSG Ratio

Glutathione/glutathione disulfide (GSH/GSSG) ratios were detected and quantified using the GSH/GSSG-Glo™ assay (Promega, Southampton, UK). Cells were pretreated with EtOAc fractions of *P. nudicaule* or *P. rhoeas* 1 h prior to LPS (5 ng/mL) treatment for 12 h. Thereafter, the media were discarded and replaced with either total glutathione lysis reagent or oxidized glutathione lysis reagent, and cells were agitated for 5 min. Both plates were kept at room temperature for 30 min before the luciferin generation reagent was added. Next, the luciferin detection reagent was introduced and incubated for 15 min, following which luminescence was measured (integration time = 0.3 s). The GSH/GSSG ratio was calculated as follows: GSH/GSSG ratio = (µM total glutathione treated − (µM GSSG-treated × 2))/µM GSSG-treated.

### 2.7. Measurement of Intracellular ROS

Intracellular ROS production was measured using 2,7-dichlorofluorescein diacetate (DCF-DA). This substrate freely permeates the cells, and upon incorporation, is oxidized to fluorescent DCF. Cells were pre-incubated with EtOAc fractions of *P. nudicaule* or *P. rhoeas* for 1 h and then incubated with LPS (5 µg/mL) for 18 h. Cells were then stained with 20 µM DCF-DA (Sigma-Aldrich, #D6883, St. Louis, MO, USA) for 30 min at 37 °C in the dark, following which they were collected, washed twice with PBS, and subjected to an analysis of a total of 10,000 events using a flow cytometer (BD Biosciences, San Jose, CA, USA). To confirm the involvement of elevated ROS in the LPS-induced inflammatory response, cells were pre-incubated with ascorbic acid (100 µM), an established antioxidant, for 1 h prior to LPS treatment.

### 2.8. Real-Time Quantitative PCR

RAW264.7 cells (5 × 10^5^ cells/well) were seeded in 6-well plates and incubated overnight. The cells were then pretreated with EtOAc fractions of *P. nudicaule* or *P. rhoeas* 1 h prior to LPS (5 or 10 ng/mL) treatment for 4 or 8 h. Isolation of total RNA using the TRI Reagent solution (Ambion, Waltham, MA, USA), its reverse transcription to cDNA using the PrimeScript first-strand cDNA synthesis kit (Takara Korea Biomedical Inc., Seoul, Republic of Korea), and amplification of each cDNA and monitoring using a Sensi FAST SYBR No-ROX kit (Bioline, Taunton, MA, USA) on a StepOnePlus instrument (Waltham, MA, USA) were previously described [28]. Specific primers used as follows: HO-1 (forward: 5′-AAGCCGAGAATGCTGAGTTCA-3′ and reverse: 5′-GCCGT GTAGATATGGTACAAGGA-3′), GCLC (forward: 5′-GTCCTCAGGTGACATTCCAAG C-3′ and reverse: 5′-TGTTCTTCAGGGGCTCCAGTC-3′), and NQO1 (forward: 5′-GGCAGAAGAGCACTGATCGTA-3′ and reverse: 5′-TGATGGGATTGAAGTTCATGGC-3′). Specific primers used for IL-6, TNF-α, IL-1β, and GAPDH were previously described [28]. Data are presented as mean ± SEM from at least three independent experiments in triplicate.

### 2.9. Preparation of Nuclear and Cytosolic Extracts

RAW264.7 cells were lysed with 200 μL of 10 mM KCl, 0.2 mM EDTA, 1.5 mM MgCl_2_, 0.5 mM DTT, and 0.2 mM phenylmethylsulfonyl fluoride (PMSF) at 4 °C for 30 min. The lysates were then centrifuged for 5 min at 14,000× *g*, and the supernatants were stored as the cytosolic extracts at −70 °C. The resulting pellets were re-suspended in 30 μL of ice-cold 20 mM HEPES (pH 7.9), 420 mM NaCl, 1.5 mM MgCl_2_, 20% (*v/v*) glycerol, 0.2 mM EDTA, 0.5 mM DTT, and 0.2 mM PMSF. After incubation at 4 °C for 30 min, the lysates were centrifuged for 10 min at 14,000× *g*, and the supernatants were stored as nuclear extracts at −70 °C. Concentrations of the cytosolic and nuclear extracts were determined using a BCA kit (Bio-Rad, Richmond, CA, USA).

### 2.10. Western Blotting Analysis

Cells were pre-treated with EtOAc fractions of *P. nudicaule* or *P. rhoeas* for 1 h and then incubated with LPS (5 ng/mL) for 30 min–24 h. Nuclear, cytoplasmic, and whole-cell extracts were prepared, and western blotting was performed as described previously [29]. Primary antibodies against Nrf2, lamin B, cyclooxygenase (COX) 2, inducible NO synthase (iNOS), p65, phospho (p)-p65, IκBα, p-IκBα, STAT3, and p-STAT3 were purchased from Cell Signaling Technology (Danvers, MA, USA). β-actin was from Sigma-Aldrich (Sigma-Aldrich, St. Louis, MO, USA). HRP-conjugated anti-mouse IgG and anti-rabbit secondary antibodies (1:5000–1:10,000) were from Jackson Immuno Research Laboratories, Inc. (West Grove, PA, USA). ImageJ software (https://imagej.nih.gov/ij/, accessed on 5 November 2021). was used for densitometric analysis of each protein band. Data are presented as mean ± Standard Deviation (SD) from at least three independent experiments.

### 2.11. Enzyme-Linked Immunosorbent Assay (ELISA) Assays

Cells (5 × 10^5^ cells/well in 6-well plates) were pre-incubated with EtOAc fractions of *P. nudicaule* or *P. rhoeas* for 1 h and incubated with LPS (5 ng/mL) for 24 h. Analysis of PEG2 secretion using an ELISA kit for PGE2 (Biochem Inc., Farmingdale, NY, USA) was performed according to the manufacturer’s instructions and as described previously [28]. Data are presented as mean ± SEM from at least three independent experiments in triplicate.

### 2.12. Statistical Analysis

Every experiment was performed at least three independent times in triplicate. SigmaPlot 10.0 software (Systat Software, Inc., San Jose, CA, USA) was used to perform an unpaired Student’s *t*-test between control and LPS treatment or LPS alone and co-treatment of EtOAc fractions or EtOH extracts of *P. nudicaule* and *P. rhoeas* with LPS to calculate the *p*-value. *p* < 0.05 was used as a statistically significant level.

### 2.13. Investigation and Identification of Bioactivity-Specific Metabolites

To investigate the anti-inflammatory and antioxidant properties of phytochemicals, liquid chromatography coupled with quadrupole time-of-flight mass spectrometry-based alkaloid profiling of EtOH extracts and EtOAc fractions was performed using chromatographic analysis, as previously reported [22]. Profiling data acquired from 12 samples, including EtOH extracts and EtOAc fractions of *P. nudicaule* and *P. rhoeas* in the untargeted scan were processed using MarkerView 1.3.1 (SCIEX, Foster City, CA, USA). To correlate alkaloid profiles and associated biological data of all extracts and fractions, biochemometric analysis was performed, where processed metabolites data including *m*/*z*, retention time, and peak area were merged with each of the bioactive data of NO, ROS, and PGE2 evaluations to form the final matrix for the investigation of bioactivity-specific metabolites. The Pearson method was used for metabolite and biological activity correlation analyses. The significant differences between the EtOAc and EtOH groups were evaluated using a t-test with Bonferroni correction. All statistical analyses were performed using R software (version 4.1.0; http://cran.r-project.org/bin/windows/base/old/4.1.0, accessed on 5 November 2021; R Foundation for Statistical Computing, Vienna, Austria). Mass spectrometric identification was performed in positive ion mode using PeakView and MasterView software (SCIEX, Foster City, CA, USA) and molecular networking analysis.

## 3. Results

### 3.1. EtOAc Fractions of P. nudicaule and P. rhoeas Reduce the LPS-Induced NO and PGE2

**Figure 1:**
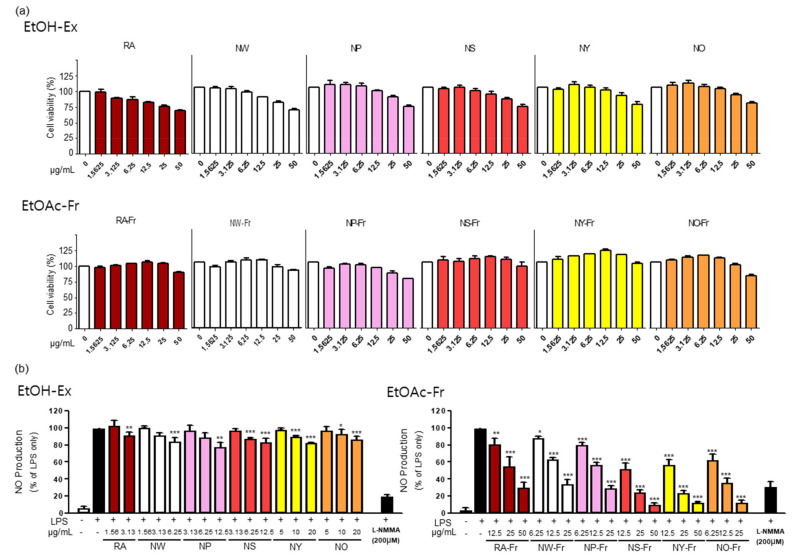
Effects of *P. nudicaule* and *P. rhoeas* extracts and ethyl acetate (EtOAc) fraction on cytotoxicity and lipopolysaccharides (LPS)-induced nitric oxide (NO) production. (**a**) RAW264.7 cells were treated with each ethanol (EtOH) extract and EtOAc fraction of *P. nudicaule* and *P. rhoeas* (1.5625–50 μg/mL) for 24 h. Cell viability was examined by 3-(4,5-dimethylthiazol-2-yl)- and 5-diphenyl-tetrazolium bromide (MTT) assays. (**b**) RAW264.7 cells were treated with each EtOH extract and the EtOAc fraction of *P. nudicaule* and *P. rhoeas* as indicated, and LPS (5 ng/mL) for 24 h. NO production was examined in the culture supernatants. Data are presented as mean ± standard error of the mean (SEM) from at least three independent experiments in triplicate. * *p* < 0.05, ** *p* < 0.01 and *** *p* < 0.001 imply statistically significant differences compared with the LPS only treated group.

To investigate the potential antioxidant and anti-inflammatory effects of *P. nudicaule* and *P. rhoeas*, we first checked the cytotoxicity of each EtOH extract and EtOAc fraction of both plants in RAW264.7 cells. As shown in Figure 1a, the EtOAc fractions of the plants were generally less toxic than their corresponding EtOH extracts. The EtOAc fractions were not cytotoxic up to 25 μg/mL (NW-Fr, NP-Fr, and NO-Fr) and 50 μg/mL (RA-Fr, NS-Fr, NY-Fr), whereas the EtOH extracts were harmless up to 3.125 μg/mL (RA), 6.25 μg/mL (NW), and 12.5 μg/mL (NP, NS, NY, and NO). Based on these results, the non-toxic concentrations of each EtOH extract and EtOAc fraction of the plants were used for further investigation.

Furthermore, antioxidant and anti-inflammatory effects of EtOH extracts and EtOAc fractions of *P. nudicaule* and *P. rhoeas* were evaluated by measuring their inhibitory effects on LPS-induced NO production in RAW264.7 cells, and the results suggest the better performance of EtOAc fractions of *P. nudicaule* and *P. rhoeas* than of EtOH extracts. As shown in Figure 1b, every EtOH extract of *P. nudicaule* and *P. rhoeas* showed a statistically significant but marginal effect on reducing LPS-mediated NO production. In contrast, EtOAc fractions of *P. nudicaule* and *P. rhoeas* significantly reduced LPS-induced NO production in a dose-dependent manner, and the inhibitory effect of every cultivar of *P. nudicaule* was better than that of *P. rhoeas* (Figure 1b). In addition, the EtOAc fraction of NO (NO-Fr) among the five cultivars of *P. nudicaule* had the best inhibitory effect (86.8% inhibition at 25 µg/mL) of LPS-induced NO production in RAW264.7, and NS-Fr (75.5% inhibition at 25 µg/mL) and NY-Fr (75.8% inhibition at 25 µg/mL) were slightly better than the other two. Thus, RA-Fr, NO-Fr, and NS-Fr were selected and used to further investigate the antioxidant and anti-inflammatory effects of *P. nudicaule* and *P. rhoeas*.

**Figure 2:**
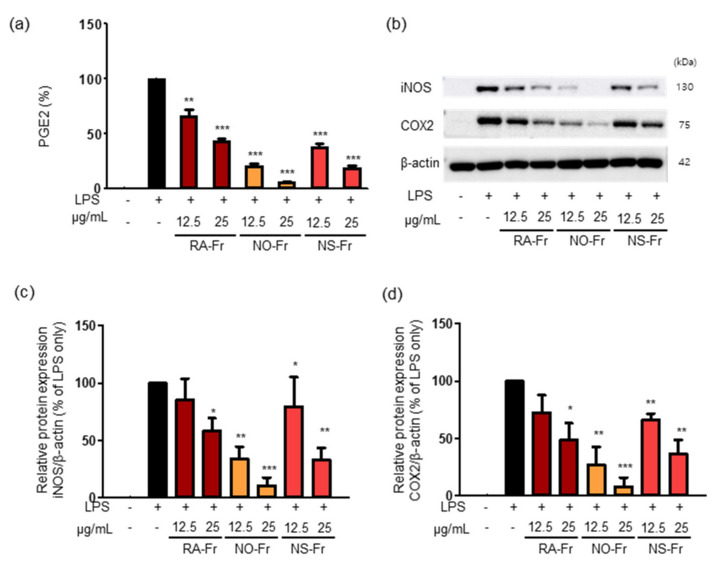
Effects of *Papaver nudicaule* orange (NO-Fr), *Papaver nudicaule* scarlet (NS-Fr) and *Papaver rhoeas* red (RA-Fr) on the expression of PGE2, iNOS and COX2. RAW264.7 cells were treated with NO-Fr, NS-Fr and RA-Fr (12.5 and 25 μg/mL), and lipopolysaccharides (LPS) (5 ng/mL) for 24 h. (**a**) The PGE2 secretion was analyzed, and data are shown as mean ± standard error of the mean (SEM). (**b**) The expression of iNOS, COX2 and β-actin in the treated cells was investigated by western blotting. (**c**,**d**) Quantification of iNOS, COX2 and β-actin expression was performed using the ImageJ software. Expression levels of iNOS and COX2 were normalized to the β-actin protein levels. Data are shown as mean ± standard deviation (SD). * *p* < 0.05, ** *p* < 0.01 and *** *p* < 0.001 imply statistically significant differences compared with the LPS only treated group.

Since iNOS is responsible for certain cytokine- or microbial product-mediated NO production and NO is known to increase prostaglandin production via the COX pathway in macrophages [30,31], we examined the effects of RA-Fr, NO-Fr, and NS-Fr on the LPS-induced expression of PGE2, COX2, and iNOS in RAW264.7 cells. As shown in Figure 2a, all EtOAc fractions significantly reduced LPS-induced PGE2 in a dose-dependent manner. In addition, every fraction suppressed the LPS-induced expression of iNOS and COX2 (Figure 2b–d) as expected. In particular, NO-Fr was the most effective in reducing LPS-induced PGE2, iNOS, and COX2, similar to the suppression of LPS-induced NO. Together, these results suggest that the EtOAc fractions of both *P. nudicaule* and *P. rhoeas* might be potent antioxidants and anti-inflammatory agents.

### 3.2. EtOAc Fractions of P. nudicaule and P. rhoeas Reduced LPS-Induced Inflammatory Cytokines and Inhibited the LPS-Mediated Activation of NF-κB and STAT3

**Figure 3:**
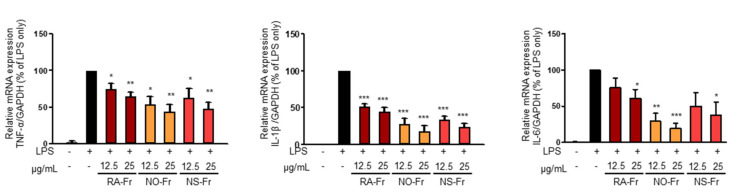
Effects of ethyl acetate (EtOAc) fraction of *Papaver rhoeas* red (RA-Fr), *Papaver nudicaule* orange (NO-Fr), *Papaver nudicaule* scarlet (NS-Fr) on the expression of TNFα, IL-1β and IL-5. RAW264.7 cells were treated with each EtOAc fraction (12.5 and 25 μg/mL) and LPS (5 ng/mL) for 4 h. The expression level of each mRNA was analyzed by normalization to the GAPDH levels. Data are shown as mean ± standard error of the mean (SEM). * *p* < 0.05, ** *p* < 0.01 and *** *p* < 0.001 imply statistically significant differences compared with the LPS only treated group.

**Figure 4:**
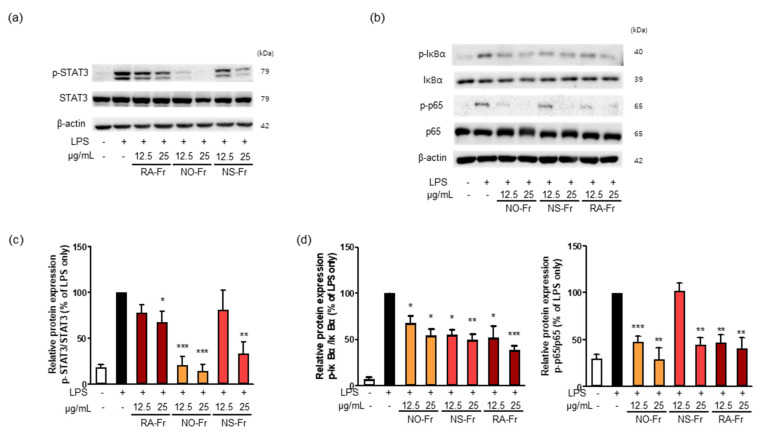
Effects of ethyl acetate (EtOAc) fraction of *Papaver nudicaule* orange (NO-Fr), *Papaver nudicaule* scarlet (NS-Fr), and *Papaver rhoeas* red (RA-Fr) on the STAT3 and NF-κB signaling pathways. (**a**,**b**) Expression and phosphorylation of STAT3 and NF-κB were examined by western blotting in RAW264.7 cells treated as indicated. (**c**) Expression levels of p-STAT3 were normalized to the STAT3 protein levels. (**d**) Expression levels of p-IκBα and p-p65 were normalized to the IκBα and p65 protein levels, respectively. Data are shown as mean ± standard deviation (SD). * *p* < 0.05, ** *p* < 0.01 and *** *p* < 0.001 imply statistically significant differences compared with the LPS only treated group.

To further elucidate the anti-inflammatory effects of EtOAc fractions of *P. nudicaule* and *P. rhoeas* and their mechanism of action, we examined mRNA expression levels of proinflammatory cytokines IL-1β, IL-6, and TNF-α. As shown in Figure 3, NO-Fr, NS-Fr, and RA-Fr effectively reduced the LPS-induced mRNA expression of IL-1β, IL-6, and TNF-α, indicating that these cultivars could regulate the expression of these genes at the transcriptional level. NF-κB and STAT3 are transcription factors that regulate the expression of the proinflammatory cytokines as well as COX2 and iNOS [28,32,33]. In addition, TNF-α and IL-6 activate NF-κB and STAT3 signaling pathways [34]. Thus, we investigated the effects of NO-Fr, NS-Fr, and RA-Fr on LPS-induced activation of NF-κB and STAT3. As shown in Figure 4a,c, the aforementioned cultivars suppressed the LPS-induced phosphorylation levels of STAT3, but had no effect on the expression of STAT3, indicating EtOAc fractions of the plant-mediated inactivation of STAT3. They also reduced the LPS-induced phosphorylation levels of IκBα and p65, indicating EtOAc fractions of the plant-mediated inactivation of NF-κB. These results suggest that the EtOAc fractions of both *P. nudicaule* and *P. rhoeas* exert their anti-inflammatory effects in LPS-stimulated macrophage cells by inhibiting NF-κB and STAT3.

### 3.3. EtOAc Fractions of P. nudicaule and P. rhoeas Reduce Oxidative Stress

**Figure 5:**
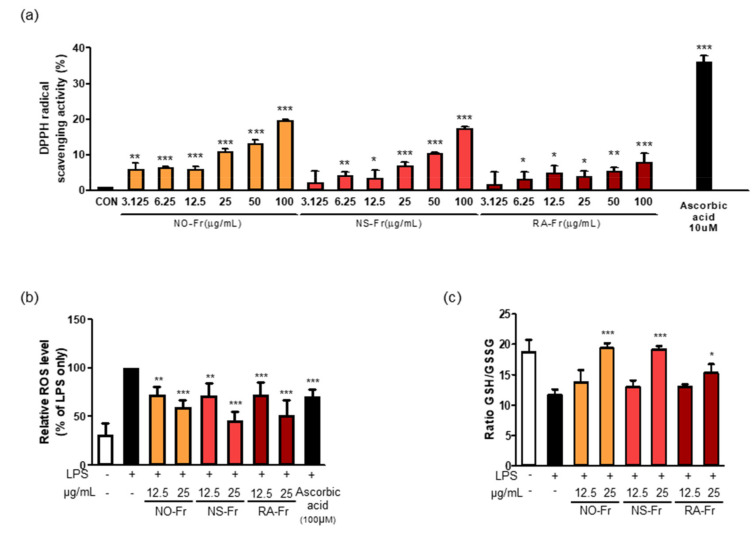
Effects of ethyl acetate (EtOAc) fraction of *Papaver nudicaule* orange (NO-Fr), *Papaver nudicaule* scarlet (NS-Fr), and *Papaver rhoeas* red (RA-Fr) on free radicals, intracellular reactive oxygen species (ROS) and Glutathione/glutathione disulfide (GSH/GSSG) ratio. (**a**) 2,2-diphenyl-1-picrylhydrazyl (DPPH) radical scavenging test by treatment with various concentrations of NO-Fr, NS-Fr and RA-Fr (μg/mL) and ascorbic acid (10 μM) as a positive control. * *p* < 0.05, ** *p* < 0.01 and *** *p* < 0.001 imply statistically significant differences compared with the control group. (**b**) Cells were treated with NO-Fr, NS-Fr and RA-Fr at two concentrations (12.5 and 25 μg/mL). After treatment for 18 h, ROS generation was measured by DCFH-DA staining with flow cytometry analysis. A graph as a percentage based on lipopolysaccharides (LPS) using mean value. (*c*) Cells were treated with NO-Fr, NS-Fr and RA-Fr at two concentrations (12.5 and 25 μg/mL). After treatment for 12 h, and the luminescence was measured. The GSH/GSSG ratio is calculated as follows: ratio GSH/GSSG treated = (µM total glutathione treated − (µM GSSG treated × 2))/µM GSSG treated. * *p* < 0.05, ** *p* < 0.01 and *** *p* < 0.001 imply statistically significant differences compared with the LPS only treated group.

Oxidative stress occurs as a result of an imbalance between generated reactive metabolites, also known as ROS, and the body’s antioxidant system. ROS, such as superoxides, hydrogen peroxide, and hydroxyl free radicals, are constantly produced in aerobic organisms. Elevated levels of ROS and free radical oxidative damage boost the generation of proinflammatory conditions and cause disease progression [10,33]. For this reason, we next investigated the potential antioxidant activities of NO-Fr, NS-Fr, and RA-Fr by measuring DPPH free radical scavenging activity. As shown in Figure 5a, the DPPH radical scavenging activity increased up to approximately 35% by ascorbic acid, a positive control. Although less than ascorbic acid, DPPH radical scavenging activities of NO-Fr, NS-Fr, and RA-Fr were observed in a dose-dependent manner (Figure 5a). We also examined the intracellular ROS in LPS-stimulated RAW264.7 cells to confirm the antioxidant activity of NO-Fr, NS-Fr, and RA-Fr. As shown in Figure 5b, NO-Fr, NS-Fr, and RA-Fr significantly decreased the LPS-increased intracellular ROS in a dose-dependent manner, and their activities were better than that of ascorbic acid. In addition, NO-Fr, NS-Fr, and RA-Fr remarkably recovered the decrease in the GSH/GSSG ratio lowered by LPS treatment in RAW264.7 cells, indicating a reduction in LPS-induced oxidative stress. These results indicated good anti-oxidative effects of the EtOAc fractions of both *P. nudicaule* and *P. rhoeas*.

### 3.4. EtOAc Fractions of P. nudicaule and P. rhoeas Increases the Expression of Antioxidant Regulators by Activating Nrf2

**Figure 6:**
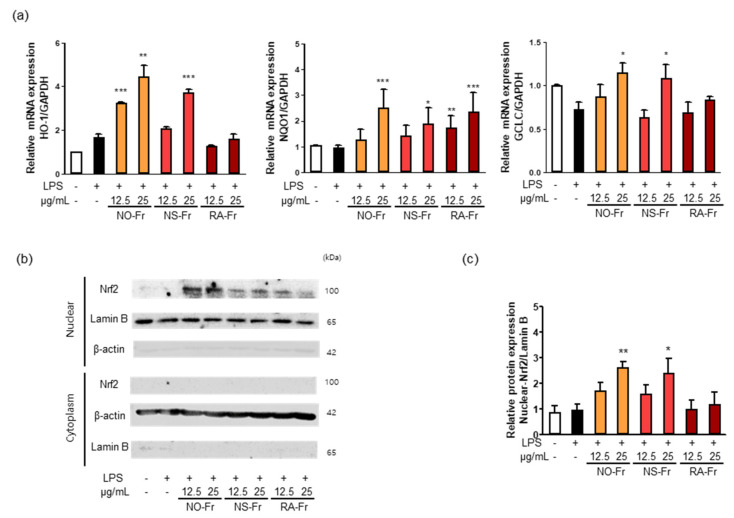
Effect of ethyl acetate (EtOAc) fraction of *Papaver nudicaule* orange (NO-Fr), *Papaver nudicaule* scarlet (NS-Fr), and *Papaver rhoeas* red (RA-Fr) on antioxidant genes (HO-1, NQO1 and GCLC) and Nrf2 signaling. RAW264.7 cells were treated with NO-Fr, NS-Fr and RA-Fr (12.5 and 25 μg/mL) and lipopolysaccharides (LPS) (10 ng/mL) for 8 h. (**a**) Expression of HO-1, NQO1 and GCLC mRNA was analyzed by normalization to the GAPDH mRNA levels. Data are shown as mean ± standard error of the mean (SEM) from at least three independent experiments in triplicate. (**b**) Nuclear translocation of Nrf2 was examined in RAW264.7 cells treated as indicated. (**c**) Expression levels of Nuclear Nrf2 shown in (**b**) were normalized to the Lamin B protein levels. Data are shown as mean ± standard deviation (SD) from at least three independent experiments. * *p* < 0.05, ** *p* < 0.01 and *** *p* < 0.001 imply statistically significant differences compared with the LPS only treated group.

Nrf2 signaling plays a vital role in intracellular antioxidant systems, including HO-1, NQO1, and GCLC [9,10,11]. Thus, we further investigated the mechanism by which NO-Fr, NS-Fr, and RA-Fr induced the antioxidant effects. As shown in Figure 6a, NO-Fr and NS-Fr increased the mRNA expression levels of HO-1, GCLC, and NQO1, and RA-Fr increased the expression of NQO1 in LPS-stimulated RAW264.7 cells. As the expression of antioxidant regulators is regulated by Nrf2 [8,9,11], we examined the expression of Nrf2 in the cytoplasm and nucleus of LPS-stimulated cells and found that NO-Fr and NS-Fr significantly increased Nrf2 expression in the nucleus but not in the cytoplasm, while RA-Fr had no effect on the expression of Nrf2 in both the cytoplasm and nucleus (Figure 6b,c). These results suggest that the EtOAc fraction of *P. nudicaule* exerts antioxidant effects via the Nrf2-mediated induction of antioxidant genes, whereas the EtOAc fraction of *P. rhoeas* had an Nrf2-independent increase in NQO1.

### 3.5. Investigation and Identification of Bioactivity-Specific Metabolites

**Figure 7:**
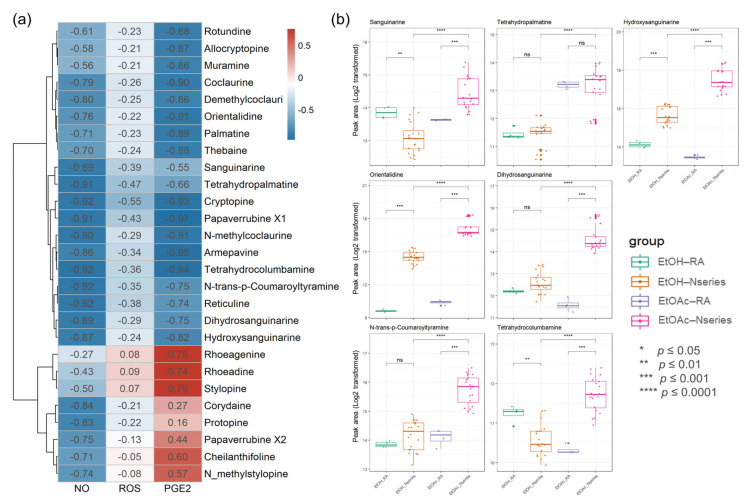
A heat map of bioactive correlation of identified metabolites from ethanol (EtOH) extracts and ethyl acetate (EtOAc) fractions of *P. nudicaule* and *P. rhoeas*. Each value in the heat map is a colored representation of a calculated Pearson correlation coefficient of metabolites with NO, ROS, and PGE2. Red and blue colors indicate an increase and decrease of Pearson correlation values, respectively (**a**). The box plots show the differential peak area of significant seven metabolites analyzed using biochemometrics (**b**). * *p* ≤ 0.05, ** *p* ≤ 0.01, *** *p* ≤ 0.001, and **** *p* ≤ 0.0001; EtOAc fraction of P. rhoeas (EtOAc-RA) vs. EtOAc fractions of *P. nudicaule* (EtOAc-N series), EtOH extract of *P. rhoeas* (EtOH-RA) vs. EtOH extract of *P. nudicaule* (EtOH-N series), and EtOH-N series vs. EtOAc-N series.

The introduction of biochemometric analyses to natural products in extracts or fractions provides an opportunity to unravel potential multiple effects of multi-components and provide potential markers in various components [35,36]. To investigate antioxidant- and anti-inflammation-specific metabolites in *P. nudicaule*, their metabolites and bioactivity (NO, PGE2, and ROS) from the EtOH extract and EtOAc fractions were linked using Pearson’s correlation and compared to those of *P. rhoeas*. The Pearson correlation coefficient between the metabolite levels in the two groups, including 12 samples, and each bioactivity was calculated. Values of r > 0.7 (or r < −0.7) confirm that a strong correlation between metabolite and bioactivity exists, and values between 0.3 and 0.7 (−0.3 and −0.7) confirm a moderate correlation, whereas values of r < 0.3 (or r > −0.3) affirm a weak correlation. The processed metabolomic data included 876 metabolites. Among them, 493 metabolites showed a strong negative correlation between the EtOAc group and inhibitory effects on LPS-induced NO production. Identifying relevant active molecules from complex mixtures represents a major challenge in natural product drug discovery [22,37,38], and identified metabolites (Table S1) were labeled on the integrated correlation to obtain potent bioactive compound information and dereplicate effectively (Figure 7a). To investigate potentially bioactive metabolites in compound mixtures from EtOAc fractions and EtOH extracts, significant metabolites represent potent metabolites with a statistically significantly strong biochemometric value (strong Pearson correlation value (r < −0.7) and significance (FDR-corrected, *p* value < 0.05)) and a higher average of peak area. Each EtOAc fraction of *P. nudicaule* and *P. rhoeas* was compared to their EtOH extracts according to the inhibitory effects on LPS-induced NO production in RAW264.7 cells. Finally, seven metabolites, including sanguinarine, tetrahydropalmatine, hydroxysanguianrine, orientalidine, dihydrosanguinarine, N-trans-*p*-coumaroyltyramine, and tetrahydrocolcumbanine were investigated and showed a strong significant correlation with inhibition of LPS-induced NO and PGE2. Sanguinarine, tetrahydropalmatine, N-trans-*p*-coumaroyltyramine, and tetrahydrocolcumbanine additionally presented a significant correlation with the reduction of ROS. Variations in the levels of significant metabolites in the samples are shown in Figure 7b. The content of metabolites in EtOAc fractions of *P. nudicaule* are relatively higher than the others, except for tetrahydropalmatine.

## 4. Discussion

Various pharmacological effects of *P. rhoeas* extracts, including antioxidant, cytoprotective, antimicrobial, antimutagenic, anti-analgesic, and antiulcerogenic activities have been uncovered [15,19,23,24,25,26,27]. However, the EtOAc fraction of the *P. rhoeas* EtOH extracts has not been evaluated for its pharmacological effects. Our recent reports presented the anti-inflammatory effects of the *P. nudicaule* EtOH extract, and metabolite profiling of *P. rhoeas* and *P. nudicaule* suggested isoquinoline alkaloids as potential pharmacologically active compounds in the plants [22,28,38]. In the current study, liquid-liquid extraction-based EtOAc fractions from EtOH extracts of *P. nudicaule* and *P. rhoeas* were prepared to enrich alkaloids. EtOAc fractions of the plants compared to their EtOH extracts showed less toxicity but more inhibitory activity against LPS-induced NO production (Figure 1). NO is produced in large quantities during inflammation and interacts with other ROS to produce reactive nitrogen oxide species [39,40]. Therefore, it can be hypothesized that the reduction of NO is involved in anti-inflammatory and antioxidant responses. EtOAc fractions of both plants lowered the LPS-induced production of proinflammatory molecules (Figure 2) and cytokines (Figure 3) and inhibited LPS-activated STAT3 and NF-κB (Figure 4). NF-κB and STAT3 are crucial transcription factors involved in immune response and inflammatory diseases by regulating the expression of inflammation-related genes, including COX2, iNOS, and inflammatory cytokines [34,41]. These results indicated that EtOAc fractionations of *P. nudicaule* and *P. rhoeas* compared to their EtOH extracts showed much better anti-inflammatory effects through inhibiting both NF-κB and STAT3, and suggest that alkaloids of the plants can be a very attractive strategy for use in inflammatory diseases.

ROS, such as superoxide, hydrogen peroxide, and hydroxyl free radicals, are constantly produced in aerobic organisms [42,43]. Hyperinflammatory responses reduce the production of antioxidant enzymes that in turn protect tissues from oxidative damage [8,11]. Nrf2 protects cells from stressors, including endogenous substances, ROS, radiation, environmental toxins, and xenobiotics from food [11]. Nrf2 accumulates in the nucleus, where it dimerizes with small Maf proteins and binds to antioxidant response element cis-regulatory sequences to trigger transcriptional expression [44]. A large number of genes have been identified as downstream targets of Nrf2, including NQO1 and HO-1. Additionally, NF-κB signaling inhibits the antioxidant effect by inhibiting the Nrf2-Keap1 pathway through the interaction of p65 and Keap1 [45,46]. EtOAc fractionations of *P. nudicaule* and *P. rhoeas* showed significant free radical scavenging activity and decreased LPS-induced reactive oxygen species and oxidized glutathione (Figure 5). EtOAc fractions of *P. nudicaule* increased the expression of HO-1, NQO-1, GCLC and nuclear Nrf2 in LPS-stimulated cells, while that of *P. rhoeas* enhanced only NQO-1 (Figure 6). Together, these results suggest that EtOAc fractions of *P. nudicaule* exert antioxidant effects via the Nrf2-mediated induction of antioxidant genes, whereas the EtOAc fraction of *P. rhoeas* had a Nrf2-independent increase in NQO1, probably by inhibiting NF-κB.

Moreover, EtOAc fractions of *P. nudicaule* compared to the EtOAc fraction of *P. rhoeas* presented better effects in both antioxidant and anti-inflammatory activity. Therefore, to identify potential candidate alkaloids involved in antioxidant and anti-inflammatory effects of *P. nudicaule*, we investigated antioxidant- and anti-inflammation-specific metabolites of *P. nudicaule* cultivars. Their metabolites (876 metabolites) and biological activities on LPS-induced NO, PGE2, and ROS production from the EtOH extract and EtOAc fractions were linked using Pearson’s correlation and also compared to those of *P. rhoeas*. Based on correlation analysis results, important metabolites analyzed using biochemometrics were selected. Among identified potent metabolites, those with changes in the sample’s significant metabolite levels are shown in Figure 7b. Among them, sanguinarine is known to have anti-inflammatory and neuroprotective effects [47,48], and tetrahydropalmatine is known to inhibit oxidative stress and inflammasome activation [49,50]. Dihydrosanguinarine is also known to inhibit pancreatic cancer cells [51]. In addition, 6-acetonyl-5,6-dihydrosanguinarine has been reported to trigger proinflammatory cytokine production [52]. However, other metabolites remain unknown. These metabolites may contribute to antioxidant and anti-inflammatory effects. Therefore, it is necessary to study the mechanism of antioxidant and anti-inflammatory effects through follow-up studies of selected novel metabolites.

## 5. Conclusions

The current study demonstrates that EtOAc fractions of *P. nudicaule* and *P. rhoeas* had lower cytotoxicity compared with their EtOH extracts and exhibited promising antioxidant and anti-inflammatory activities. Metabolomic and biochemometric analyses of the plants suggested seven potential active alkaloids were enriched in the EtOAc fraction of *P. nudicaule*. These results could serve as the rationale for further investigation of EtOAc fractions of the plants and alkaloids for the development of therapeutic interventions for inflammation-related diseases.

## Figures and Tables

**Table 1:** *Papaver rhoeas*, five cultivars of *Papaver nudicaule* with different flower colors and abbreviations used.

Species	Flower Color	Abbreviation of Ethanolic Extract	Abbreviation of EtOAc Fraction From Each Ethanolic Extracts
*Papaver rhoeas*	Red	RA	RA-Fr
*Papaver nudicaule*	White	NW	NW-Fr
*Papaver nudicaule*	Pink	NP	NP-Fr
*Papaver nudicaule*	Scarlet	NS	NS-Fr
*Papaver nudicaule*	Yellow	NY	NY-Fr
*Papaver nudicaule*	Orange	NO	NO-Fr

## Data Availability

Data is contained within the article and Supplementary Materials.

## Supplementary Materials

The following are available online at https://www.mdpi.com/article/10.3390/antiox10121895/s1, Table S1: Identification of chemical components authentically or tentatively in EtOH extracts and EtOAc fractions of *P. nudicaule* and *P. rhoeas* from using LC-ESI-QTOF MS/MS.
