# Supplementary material for: Ethyl Acetate Fractions of Papaver rhoeas L. and Papaver nudicaule L. Exert Antioxidant and Anti-Inflammatory Activities
Source: Antioxidants (Basel). 2021 Nov 26;10(12):1895. doi: 10.3390/antiox10121895 (PMC8750608; doi:10.3390/antiox10121895)
Supplement: Supplementary file 1 [file antioxidants-10-01895-s001.zip › antioxidants-1473247-supplementary.pdf]

Table S1. Identification of chemical components authentically or tentatively in EtOH extracts and EtOAc fractions of *P. nudicaule* and *P. rhoeas* from using LC-ESI-QTOF MS/MS

No.	Name	Formula	mass (Da)	Expected RT (min)	Adduct	Found at mass (Da)	Error (ppm)	MS/MS product ions	Identified with
1	Demethylcoclaurine	C16H17NO3	271.12084	5.02	[M+H] +	272.1283	0.1	107.0495, 255.1023,161.0605, 143.0498	†
2	Armepavine	C19H23NO3	313.16779	5.60	[M+H] +	314.1743	-1.8	298.1074, 226.0860, 299.1161, 211.0748	[22]
3	Coclaurine	C17H19NO3	285.13649	6.06	[M+H] +	286.1439	0.5	107.0497, 269.1172, 175.0751, 237.0920, 209.0946	†
4	N-methylcoclaurine	C18H21NO3	300.1599	6.12	[M+H] +	300.1596	0.7	107.0499, 269.1174, 175.0755, 237.0917, 137.0593	[37]
5	Papaverrubine X1	C21H23NO6	385.1525	6.64	[M+H] +	386.1598	-0.1	368.1494, 297.1133, 206.1174, 266.0922, 223.0744	[37]
6	Reticuline	C19H23NO4	329.16271	6.74	[M+H] +	330.1696	-1.1	192.1017, 137.0582, 299.1256, 177.0804	†
7	Cheilanthifoline	C19H19NO4	325.13141	7.68	[M+H] +	326.1387	0.1	178.0859, 163.0635, 151.0742, 190.0854	[37]
8	Tetrahydrocolumbamine	C20H23NO4	341.16271	8.37	[M+H] +	342.1703	1.0	178.0869, 163.0632, 151.0755, 176.0707	†
9	Papaverrubine X2	C21H23NO6	385.1525	8.65	[M+H] +	386.1598	0.1	368.1483, 206.1170, 190.0859, 336.1223	[37]
10	Rotundine	C21H25NO4	355.17836	8.84	[M+H] +	356.1853	-1.0	192.1017, 177.0782, 148.0758	[22]
11	Cryptopine	C21H23NO5	369.1576	9.01	[M+H] +	370.1651	0.4	204.1021, 205.1102, 190.0865, 165.0914	[37]
12	Protopine	C20H19NO5	353.12632	9.25	[M+H] +	354.1337	0.2	188.0708, 189.0783, 149.0595, 275.0708	†
13	Muramine	C22H27NO5	385.18892	9.89	[M+H] +	386.1964	0.6	204.1022, 165.0909, 306.1258, 368.1865, 222.1126	[22]
14	Rhoeagenine	C20H19NO6	369.12124	10.20	[M+H] +	370.1285	0.0	352.1175, 190.0858, 188.0701, 320.0912, 321.0753	[22]
15	Allocryptopine	C21H23NO5	369.1576	10.23	[M+H] +	370.1646	-0.7	352.1181, 190.0857, 188.0701, 320.0913, 291.0649	†
16	N_methylstylopine	C20H20NO4	338.13923	10.23	[M] +	338.1387	-0.7	190.0860, 188.0703, 149.0595, 249.0910, 189.0769	[37]
17	Tetrahydropalmatine	C21H25NO4	355.17836	10.47	[M+H] +	356.1860	0.9	192.1024, 165.0916, 176.0713, 177.0792	†
18	Rhoeadine	C21H21NO6	383.13689	10.52	[M+H] +	384.1442	-0.4	352.1192, 190.0861, 188.0705,320.0924, 303.0676	[22]
19	Stylopine	C19H17NO4	323.11576	10.85	[M+H] +	324.1231	0.2	176.0703, 149.0592, 91.0546	†
20	N-trans-p-Coumaroyltyramine	C17H17NO3	283.12084	11.34	[M+H] +	284.1282	0.1	147.0439, 121.0645, 119.0496, 91.0558	#
21	Orientalidine	C22H23NO6	397.15254	11.46	[M+H] +	398.1597	-0.4	382.1279, 336.1231, 364.1174, 193.0851	*
22	Corydaine	C22H27NO4	369.19401	12.16	[M+H] +	370.2015	0.5	192.1021, 165.0912, 150.0677, 176.0707, 354.1705	†
23	Thebaine	C19H21NO3	311.15214	12.74	[M+H] +	312.1596	0.7	252.0782, 251.1063, 223.1117, 281.1173	[38]
24	Sanguinarine	C20H14NO4	332.09228	12.85	[M] +	332.0918	-1.6	304.0960, 274.0867, 246.0924, 317.0697, 218.0958	†
25	Palmatine	C21H22NO4	352.15488	13.80	[M] +	352.1546	-1.0	336.1224, 322.1072, 308.1280, 337.1307	[38]
26	Hydroxysanguinarine	C20H13NO5	347.07937	17.70	[M+H] +	348.0868	0.5	333.0638, 305.0690, 320.0926, 98.9848	*
27	Dihydrosanguinarine	C20H15NO4	333.10011	19.03	[M+H] +	334.1068	-1.2	318.0755, 319.0832, 276.1053, 304.0965	†

† Reference standard

In-house ms/ms library and online database; such as GNPS, MASS bank or Metlin

* Monoisotope Mass
